# Polarization-State Degradation and Compensation of Folding Mirrors in Polarization-Encoded Detector-Multiplexed Infrared Imaging

**DOI:** 10.3390/s26175634

**Published:** 2026-09-04

**Authors:** Zibo Yu, Yishi Qiao, Zhenyuan Guo, Yunhan Ma, Menghan Bai, Jiaqi Wang, Chenchen Gao, Chunyu Liu

**Affiliations:** 1 Changchun Institute of Optics, Fine Mechanics and Physics, Chinese Academy of Sciences, Changchun 130033, China; yuzibo22@mails.ucas.ac.cn (Z.Y.); guozhenyuan23@mails.ucas.ac.cn (Z.G.); mayunhan22@mails.ucas.ac.cn (Y.M.); baimenghan21@mails.ucas.ac.cn (M.B.); wangjiaqi222@mails.ucas.ac.cn (J.W.); gaochenchen24@mails.ucas.ac.cn (C.G.); 2 University of Chinese Academy of Sciences, Beijing 101408, China; 3 China Siwei Surveying and Mapping Technology Co., Ltd., Beijing 100089, China; qiaoyishi@chinasiwei.com

**Keywords:** polarization encoding, detector multiplexing, infrared imaging, folding-mirror polarization distortion, Mueller-matrix compensation

## Abstract

Polarization-encoded detector multiplexing has recently shown promise for compact wide-field infrared imaging, where multiple field-of-view (FOV) regions are mapped onto a shared detector area and distinguished through Stokes-vector decoding. The practical implementation of this architecture, however, requires reflective folding components for optical path compression and detector reuse. Oblique metallic reflection introduces unequal amplitude attenuation and phase retardation between the p- and s-polarized components, which can deform the designed polarization code before it reaches the polarization-resolved detector. This work establishes a coordinate-consistent Jones–Mueller model for eight peripheral reflections from the internal octagonal mirror and one central direct path. The model distinguishes deterministic polarization-state transport from true depolarization and compares detector-side calibration, encoder pre-compensation, one shared liquid-crystal polarization retarder (LCPR), and a segmented-LCPR upper bound. Using a unified aluminum model at 4.0 um, the mirror displaces the encoded states by several degrees but does not depolarize a fully polarized monochromatic ray. Across 60 Monte Carlo trials with 2000 samples per channel, all principal methods remain approximately 100% accurate at an additive Gaussian-noise standard deviation of σ = 0.02, normalized relative to unit S_0_. At σ = 0.15, the ideal-codebook, calibrated-codebook, and pre-compensated decoders achieve 90.87%, 91.24%, and 89.87%, respectively. A shared LCPR provides little global benefit, whereas segmented settings recover individual states at the cost of channel-resolved hardware. The results show that mirror-aware calibration, code-space separation, and physically implementable compensation must be considered jointly.

## 1. Introduction

Wide-field infrared imaging systems are increasingly required to provide large spatial coverage, high sensitivity, and compact payload integration. These requirements are difficult to satisfy simultaneously because infrared focal-plane arrays are constrained by detector format, cryogenic cooling, fabrication cost, and system power consumption. Detector multiplexing offers an attractive route for improving the utilization efficiency of limited detector resources by folding signals from multiple FOV regions onto a common detector region. However, direct geometric multiplexing causes severe spatial aliasing, and intensity-only measurements generally cannot determine the original spatial source of an overlapped target [1,2,3].

In our previous work, polarization encoding was introduced as an additional physical dimension for detector-multiplexed infrared imaging [1]. The full FOV was divided into multiple sub-regions, and each region was assigned a distinct polarization state. After optical folding and detector reuse, polarization-resolved measurements were used to reconstruct Stokes vectors, and the FOV origin of each pixel or target was identified through vector similarity in the Stokes domain. This strategy extended detector multiplexing from scalar intensity coding to vector-field coding and demonstrated robust spatial reconstruction under aliased measurements [1,4,5,6,7,8,9,10,11,12].

The broader idea of expanding FOV through polarization multiplexing was demonstrated by Douglass et al. [13]. The present architecture differs in using a contiguous 3 × 3 partition and an internal octagonal reflective assembly to redirect eight peripheral sub-FOVs while retaining a direct central channel.

That framework, however, implicitly assumed that the designed polarization states were preserved during optical transport. This assumption is reasonable for an initial proof of concept, but it becomes insufficient when the architecture is moved toward an actual folded infrared optical system. Compact detector-sharing layouts typically require one or more reflective mirrors to redirect beams from different FOV channels onto the shared detector. At oblique incidence, a metallic folding mirror is not a polarization-neutral component. The p- and s-polarized field components acquire different complex Fresnel reflection coefficients, leading to polarization rotation, ellipticity generation, and displacement of the encoded Stokes vectors. If this transformation is not modeled, the reference polarization codes used in the decoder no longer match the states actually measured at the detector [14,15,16,17,18,19,20].

Polarization aberrations of reflective systems have long been studied through polarization ray tracing and fold-mirror balancing [14,15,16,17,18,19,20]. More recent work emphasizes system-level coating, geometry, and analyzer calibration effects [21,22]. Unlike those general studies, the present work links the channel-dependent mirror response directly to the classification margin of a nine-region polarization codebook.

This issue is not a secondary optical detail; it directly affects the core discriminative mechanism of polarization-encoded multiplexing. The classification margin between FOV channels depends on the angular separation of their Stokes vectors. Mirror-induced distortion can move the encoded states closer together, alter the handedness or ellipticity of selected channels, and introduce angle-dependent deviations across the folded optical path. Consequently, a polarization-encoded detector-multiplexing system must include a forward model of the reflective relay optics and, when necessary, a compensation mechanism.

The present study addresses this missing link. We develop a polarization transfer model for oblique metallic reflection, derive its influence on Stokes-vector encoding, and evaluate two compensation routes. The first route is pre-compensation, in which the incident polarization state is deliberately modified before reflection so that the reflected state matches the desired code. The second route is post-compensation, in which a tunable LCPR is placed after the mirror and optimized to restore the target state. Compared with our previous system-level multiplexing study, the present work focuses on the physical integrity of the polarization channel itself.

**Figure 1:**
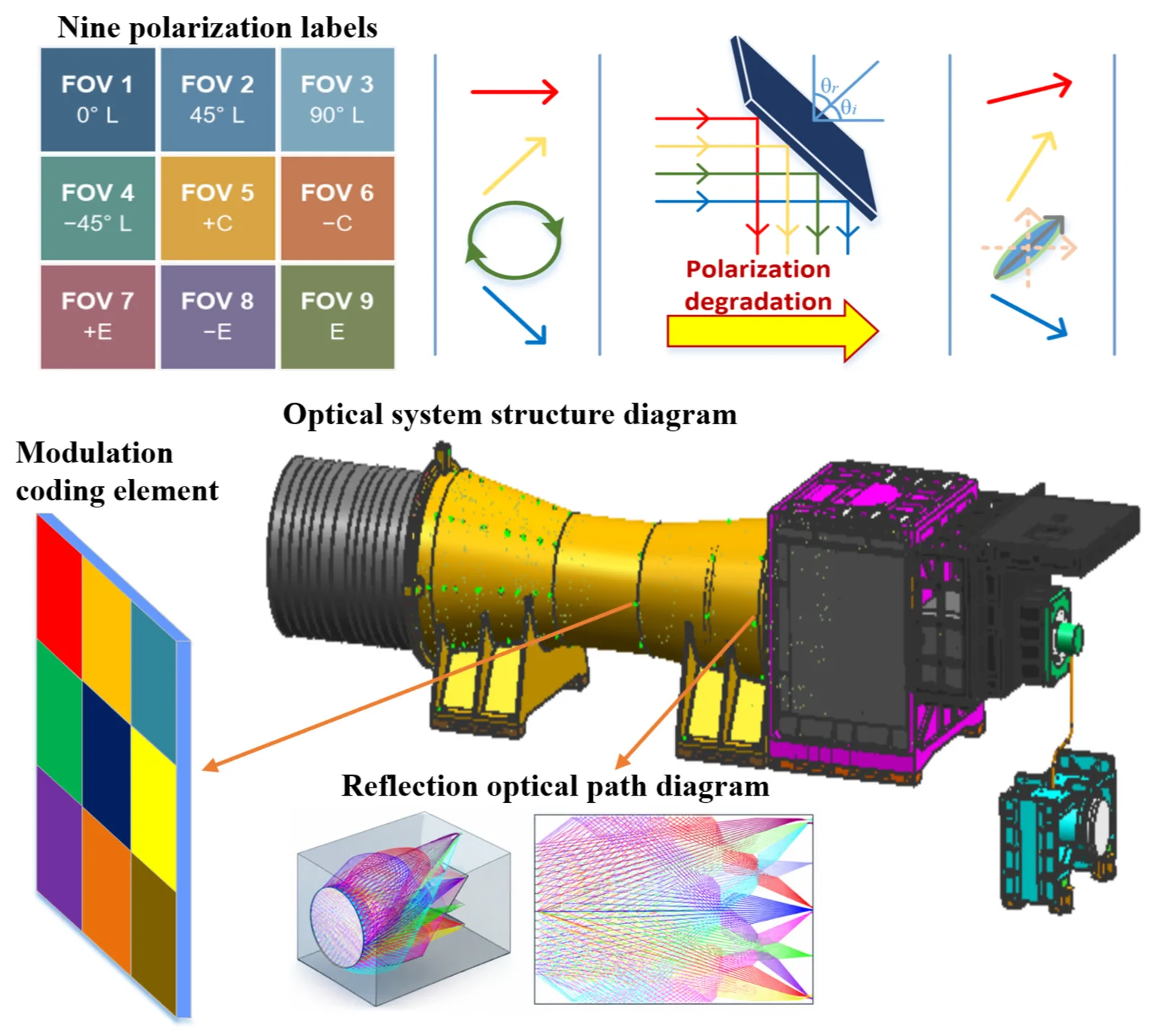
Complete nine-region polarization-encoded detector-multiplexed infrared imaging architecture. A sparse unpolarized point target is located in the 3 × 3 FOV, and every encoder tile contains a polarizer followed by its assigned polarization modulator. Eight peripheral channels are redirected by the eight internal mirror facets with channel-dependent incidence-plane azimuths, whereas channel 5 follows the central direct path.

The principal innovations are therefore: (i) a full-Stokes formulation that distinguishes deterministic state transport from true depolarization; and (ii) a fair comparison of ideal-codebook decoding, detector-side calibration, encoder pre-compensation, one shared LCPR, and a segmented-LCPR upper bound. The complete nine-region architecture and the role of the internal octagonal folding mirror are illustrated in Figure 1.

## 2. Mirror-Induced Polarization Degradation Model

### 2.1. Polarization Transfer in Detector-Multiplexed Folding Optics

In polarization-encoded detector multiplexing, each FOV region is assigned a reference Stokes vector that acts as a physical label for spatial origin. Let the kth region be encoded by

(1) $$S_{k}=[S_{0},\;S_{1},\;S_{2},\;S_{3}{]}^{T}$$

For a fully polarized state, the normalized Stokes vector can be parameterized by the polarization azimuth psi and ellipticity angle chi,

(2) $$\frac{1}{S_{0}}\left[\begin{array}{c} S_{1}\\ S_{2}\\ S_{3} \end{array}\right]=\left[\begin{array}{c} \cos(2\psi )\cos(2\chi )\\ \sin(2\psi )\cos(2\chi )\\ \sin(2\chi ) \end{array}\right]$$

In an ideal polarization-encoded multiplexing model, the detector receives an aliased intensity distribution while the Stokes vector remains a faithful marker of the source FOV. In a folded optical path, the encoded Stokes vector must instead be propagated through the mirror Mueller matrix:

(3) $$S_{k}^{det}=M_{m}\left(\theta_{k}\right)\;S_{k}^{enc}$$

where $\theta_{k}\;$ is the incidence angle associated with the kth folded channel, $S_{k}^{enc}$ is the intended encoded state, and $S_{k}^{det}$ is the state arriving at the polarimetric detector. Therefore, the actual decoding reference should be the transported state $M_{m}(\theta_{k})\;S_{k}^{enc}$, not the originally assigned ideal code.

For a linear analyzer with transmission axis beta, the measured intensity is

(4) $$I\left(\beta \right)=\frac{1}{2}(S_{0}+S_{1}\cos2\beta +S_{2}\sin2\beta )$$

and the circular channels can be written as

(5) $$I_{R}=\frac{1}{2}\left(S_{0}+S_{3}\right)$$

(6) $$I_{L}=\frac{1}{2}\left(S_{0}-S_{3}\right)$$

These expressions show that any mirror-induced variation in $S_{1}$, $S_{2}$, or $S_{3}$ directly changes the multi-channel measurements used for Stokes reconstruction. The mirror is therefore part of the encoding-decoding operator, rather than a passive relay element.

Figure 1 summarizes this physical role of the folding mirror in the detector-multiplexing chain. The key difference from the ideal encoding model is that the polarization label is no longer transported unchanged; instead, it is acted on by the mirror Mueller matrix before reaching the detector-side Stokes decoder.

For the actual 3 × 3 layout, channels 1–4 and 6–9 are reflected by eight azimuthally distributed facets, while channel 5 is transmitted through the central path. At the nominal symmetric geometry, the peripheral incidence angle is 64 deg and the incidence-plane azimuths are 135, 90, 45, 180, 0, 225, 270, and 315 deg. These channel-dependent basis rotations are included explicitly in the nine-region simulation.

### 2.2. Jones Model of Oblique Metallic Reflection

Consider a metallic folding mirror separating an incident medium with refractive index $n_{1}$ and a metal or coated reflector with complex refractive index

(7) $${\tilde{n}}_{2}=n+ik$$

For an incidence angle θ, the complex transmission angle is determined by Snell’s law. Defining

(8) $$q\left(\theta \right)=\sqrt{{\tilde{n}}_{2}^{2}-n_{1}^{2}\sin^{2}\theta }$$

the Fresnel reflection coefficients may be written as [23,24]

(9) $$r_{s}\left(\theta \right)=\frac{n_{1}\cos\theta -q(\theta )}{n_{1}\cos\theta +q(\theta )}$$

(10) $$r_{p}\left(\theta \right)=\frac{{\tilde{n}}_{2}^{2}\cos\theta -n_{1}\;q(\theta )}{{\tilde{n}}_{2}^{2}\cos\theta +n_{1}\;q(\theta )}$$

Equivalently, using the complex transmitted angle $\theta_{t}$,

(11) $$r_{s}=\frac{n_{1}\cos\theta -{\tilde{n}}_{2}\cos\theta_{t}}{n_{1}\cos\theta +{\tilde{n}}_{2}\cos\theta_{t}}$$

(12) $$r_{p}=\frac{{\tilde{n}}_{2}\cos\theta -n_{1}\cos\theta_{t}}{{\tilde{n}}_{2}\cos\theta +n_{1}\cos\theta_{t}}$$

In the p/s basis, the mirror Jones matrix is diagonal:

(13) $$J_{m}=\left[\begin{array}{cc} r_{p} & 0\\ 0 & r_{s} \end{array}\right]$$

If the laboratory x-y basis is not aligned with the p/s basis, a rotation must be included:

(14) $$J_{m,\mathrm{lab}}\left(\theta ,\gamma \right)=R^{-1}\left(\gamma \right)\;J_{m}\left(\theta \right)\;R\left(\gamma \right)$$

where γ is the angle between the laboratory x-axis and the p-polarization direction. In the current numerical model, the plane of incidence is assumed to be the x-z plane, so p is aligned with x and s is aligned with y, giving γ = 0.

Because reflection reverses the propagation direction, the incident and reflected transverse bases are transported as right-handed frames before Stokes vectors are compared. This coordinate step removes apparent sign or 90 deg changes that arise only from inconsistent p/s basis definitions; the material diattenuation and retardance remain unchanged.

The polarization distortion originates from the fact that $r_{p}$ and $r_{s}$ have both different magnitudes and different phases. Let

(15) $$r_{p}=|r_{p}|e^{i\phi_{p}},\;r_{s}=|r_{s}|e^{i\phi_{s}}$$

The mirror can then be understood as a diattenuating retarder characterized by the amplitude ratio [25]

(16) $$\eta \left(\theta \right)=\frac{|r_{p}(\theta )|}{|r_{s}(\theta )|}$$

and the differential phase

(17) $$\Delta \left(\theta \right)=\phi_{p}\left(\theta \right)-\phi_{s}\left(\theta \right)$$

The amplitude ratio changes the relative strengths of the two orthogonal linear components, while the phase difference converts a linear state into an elliptical state. This is the fundamental mechanism by which a folding mirror degrades a polarization code.

Within the present ideal model, a smooth and spatially uniform mirror is a nondepolarizing Jones element: it displaces a fully polarized state on the Poincare sphere but does not reduce its degree of polarization. True depolarization can arise after averaging over roughness, oxide or coating nonuniformity, wavelength, incidence angle, pupil position, or temporal fluctuations. The term degradation is therefore used here for code-state mismatch and possible loss of separation, not automatically for a reduction in degree of polarization.

### 2.3. Analytical Stokes Degradation of a 45 Deg Linear Code

The 45 deg linear polarization state is important because it is one of the canonical reference states used in the nine-region polarization encoding configuration. Its Jones vector in the aligned p/s basis is

(18) $$E_{in}=\frac{1}{\sqrt{2}}\left[\begin{array}{c} 1\\ 1 \end{array}\right]$$

After mirror reflection,

(19) $$E_{r}=\frac{1}{\sqrt{2}}\left[\begin{array}{c} r_{p}\\ r_{s} \end{array}\right]$$

Substituting this field into the Stokes definitions gives

(20) $$S_{0}=|r_{p}{|}^{2}+|r_{s}{|}^{2}$$

(21) $$S_{1}=|r_{p}{|}^{2}-|r_{s}{|}^{2}$$

(22) $$S_{2}=2|r_{p}||r_{s}|\cos\Delta$$

(23) $$S_{3}=2|r_{p}||r_{s}|\sin\Delta$$

The normalized reflected Stokes components are therefore

(24) $$\frac{S_{1}}{S_{0}}=\frac{\eta^{2}-1}{\eta^{2}+1}$$

(25) $$\frac{S_{2}}{S_{0}}=\frac{2\eta \cos\Delta }{\eta^{2}+1}$$

(26) $$\frac{S_{3}}{S_{0}}=\frac{2\eta \sin\Delta }{\eta^{2}+1}$$

These equations make the degradation mechanism explicit. If η = 1 and Δ = 0, the mirror preserves the 45 deg linear state. If eta differs from unity, $S_{1}$ is introduced and the linear polarization azimuth shifts. If Δ differs from zero, $S_{3}$ appears and the reflected state becomes elliptical. For metallic mirrors at large incidence angles, both effects are generally present, so the reflected Stokes vector no longer coincides with the originally assigned code.

The polarization azimuth and ellipticity angle of the reflected state can be recovered from

(27) $$\psi =\frac{1}{2}\tan^{-1}\left(\frac{S_{2}}{S_{1}}\right)$$

(28) $$\chi =\frac{1}{2}\sin^{-1}\left(\frac{S_{3}}{S_{0}}\right)$$

This analytical result also explains why a compensation element must supply an opposite effective retardance and, in some cases, a small correction to the relative p/s amplitude balance.

### 2.4. Jones-to-Mueller Conversion and Code-Space Distortion

The detector-side reconstruction and FOV classification operate in the Stokes domain, so the Jones reflection model must be converted into a Mueller matrix. Using the Pauli-like basis matrices [26,27]

(29) $$\sigma_{0}=\left[\begin{array}{cc} 1 & 0\\ 0 & 1 \end{array}\right]$$

(30) $$\sigma_{1}=\left[\begin{array}{cc} 1 & 0\\ 0 & -1 \end{array}\right]$$

(31) $$\sigma_{2}=\left[\begin{array}{cc} 0 & 1\\ 1 & 0 \end{array}\right]$$

(32) $$\sigma_{3}=\left[\begin{array}{cc} 0 & -i\\ i & 0 \end{array}\right]$$

The Mueller matrix corresponding to a Jones matrix J is

(33) $$M_{ij}=\frac{1}{2}Tr\left(\sigma_{i}J\sigma_{j}J^{†}\right)$$

This conversion is useful because the similarity-based decoder compares normalized Stokes vectors. For the i th and j th encoded FOV channels, the ideal code-space similarity is

(34) $$\rho_{ij}=\frac{s_{i}\cdot s_{j}}{∥s_{i}∥\;∥s_{j}∥}$$

where s = ${{[S}_{1},\;S_{2},\;S_{3}]}^{T}$/$S_{0}$. After mirror propagation, the full four-component Stokes vector is propagated first and normalized by the output intensity:

(35) $${S_{i}}^{\prime }=\;M_{i}S_{i},\;\;\;\;{S_{i}}^{\prime }=\frac{{\left[{S^{\prime }}_{i1},{S^{\prime }}_{i2},{S^{\prime }}_{i3}\right]}^{T}}{{S^{\prime }}_{i0}}$$

The detector-side similarity is subsequently evaluated as

(36) $${\rho^{\prime }}_{ij}=\frac{{{s_{i}}^{\prime }}^{T}{s_{j}}^{\prime }}{\left|\left|{s_{i}}^{\prime }\right|\right|\left|\left|{s_{j}}^{\prime }\right|\right|}$$

If the mirror transformation reduces the angular separation between two encoded states, their similarity increases and the probability of FOV misclassification rises under noise or modulation error. Thus, preserving polarization codes is equivalent to preserving sufficient separation on the Poincare sphere after the complete optical transfer path.

## 3. Polarization Compensation Methodology

The preceding section establishes that the folding mirror changes the polarization code through a deterministic Jones–Mueller operator. The next step is therefore not merely to observe the degradation, but to define how the original code can be recovered in a controlled optical system. Two complementary strategies are considered. Pre-compensation modifies the polarization state before reflection, so the mirror itself becomes part of the encoder. Post-compensation keeps the incident code unchanged and adds a corrective optical element after reflection. These two strategies correspond to different implementation constraints and are therefore both relevant for detector-multiplexed infrared instruments.

### 3.1. Pre-Compensation by Inverse Mueller Propagation

Pre-compensation aims to find an incident state that becomes the desired code after mirror reflection. For a target detector-side Stokes vector $S_{des}$, the required input state is formally

(37) $$S_{in}=M_{m}^{-1}S_{des}$$

For the 45 deg target state,

(38) $$S_{des}=\left[\begin{array}{c} S_{0}\\ 0\\ S_{0}\\ 0 \end{array}\right]$$

The inverse solution must satisfy the physical realizability condition

(39) $$S_{0}^{2}\geq S_{1}^{2}+S_{2}^{2}+S_{3}^{2}$$

When the computed vector is slightly outside the feasible set because of numerical conditioning or normalization choices, the polarization components can be projected onto the unit Poincare sphere for a fully polarized implementation:

(40) $$\left[\begin{array}{c} S_{1}\\ S_{2}\\ S_{3} \end{array}\right]\leftarrow \frac{S_{0}}{\sqrt{S_{1}^{2}+S_{2}^{2}+S_{3}^{2}}}\left[\begin{array}{c} S_{1}\\ S_{2}\\ S_{3} \end{array}\right]$$

The corresponding experimental control parameters are then obtained from

(41) $$\psi_{\mathrm{pre}}=\frac{1}{2}\tan^{-1}\left(\frac{S_{2}}{S_{1}}\right),\chi_{\mathrm{pre}}=\frac{1}{2}\sin^{-1}\left(\frac{S_{3}}{S_{0}}\right)$$

This strategy is particularly suitable when the polarization modulator at the intermediate image plane can be redesigned or programmed according to the known folding geometry.

For fully polarized target states, the equivalent Jones-domain inverse provides a direct realizable solution and avoids applying a 4 × 4 Mueller matrix to a three-component normalized vector. In either formulation, the full four-component Stokes vector is propagated first and normalized only afterward.

### 3.2. LCPR-Based Post-Compensation

Post-compensation keeps the incident polarization code unchanged and inserts a corrective retarder after the folding mirror. The Jones matrix of a retarder with fast-axis angle α and retardance Δ is [28,29,30,31,32]

(42) $$J_{r}=R\left(-\alpha \right)\left[\begin{array}{cc} e^{-i\delta /2} & 0\\ 0 & e^{i\delta /2} \end{array}\right]R\left(\alpha \right)$$

with $R(\alpha )=\left[\begin{array}{cc} \cos\alpha & -\sin\alpha \\ \sin\alpha & \cos\alpha \end{array}\right]$, the compensated field is

(43) $$E_{out}=J_{r}J_{m}E_{in}$$

Because absolute optical throughput is not the primary criterion for polarization-code recovery, the optimization is performed up to a complex scalar c. The Jones-domain loss function is defined as

(44) $$L\left(\alpha ,\;\delta \right)={\left|\left|Eout-cEtarget\right|\right|}^{2}$$

where $c=\frac{E_{target}^{†}E_{out}}{E_{target}^{†}E_{target}}$, the optimal compensator parameters are therefore

(45) (α ∗, δ ∗)=argmin L(α, δ)

For a mirror that primarily acts as a differential retarder between p and s components, the optimal LCPR is expected to behave close to a half-wave retarder with an angle selected to rotate the reflected polarization ellipse back toward the desired state. This expectation is consistent with the numerical results reported below.

The optimization in this subsection describes one channel or one incidence condition. After the nine optical paths have merged, one physical LCPR must apply the same fast-axis angle and retardance to every channel. Accordingly, the system simulation distinguishes a jointly optimized shared LCPR from independent per-channel settings; the latter represents a segmented or channel-separated hardware upper bound rather than one conventional element.

## 4. Simulation Results and Analysis

### 4.1. Numerical Implementation

The theoretical model was evaluated in MATLAB R2025b using a unified aluminum mirror parameter at 4.0 um. The complex refractive index was set to n+ki=6.77+38.68i, obtained by interpolation from the aluminium optical constants reported by Rakic et al. [33] and cross-checked against additional optical-constant sources [34,35]. This single material setting was used consistently for the inverse-Mueller pre-compensation simulation, the LCPR post-compensation simulation, and the nine-region FOV decoding comparison. The incidence angle was swept from 58 deg to 70 deg with a 1 deg interval for the single-state analysis.

For LCPR-based post-compensation, the incident field was fixed at 45 deg linear polarization, and the LCPR retardance and fast-axis angle were searched over discrete 2 deg intervals. To provide a direct system-level comparison of the three decoding conditions, an additional nine-region simulation was performed using the same aluminum mirror parameter. The nine FOV regions were assigned the same polarization codebook used in the previous detector-multiplexing study, including four linear states, two circular states, and three elliptical states. In the nominal nine-region geometry, the eight peripheral regions use a common 64 deg incidence angle with different incidence-plane azimuths, and the center region follows a direct path. Six conditions are evaluated under the same codebook and noise realization: ideal propagation, mirror plus ideal codebook, mirror plus a calibrated detector-side codebook, inverse pre-compensation, one jointly optimized shared LCPR, and independent segmented-LCPR settings as an upper bound. This expanded comparison preserves the original compensation study while separating physical compensation from avoidable reference-library mismatch [1]. Table 1 and Table 2 report the complete numerical protocol and channel assignments.

To keep the study physically focused, the numerical verification is first performed on the 45 deg linear code. This state is not chosen because the full encoding problem contains only one state, but because it is a canonical Stokes vector with $S_{1}\;$= 0, $S_{2}\;$=${\;S}_{0}$, and $S_{3}\;$= 0, making mirror-induced azimuth drift and ellipticity generation especially transparent. Once the single-state transfer and compensation behavior is validated, the same Mueller operator can be applied to the complete multi-state FOV codebook.

### 4.2. Mirror-Induced Degradation of the 45 Deg Polarization Code

The 45 deg linear state is designed to have $S_{1}$ = 0, $S_{2}$ = $S_{0}$, and $S_{3}$ = 0. After oblique reflection, however, the p/s amplitude imbalance and phase difference introduce nonzero $S_{1}$ and $S_{3}$ components. The generated $S_{1}$ component corresponds to a shift away from the ideal 45 deg azimuth, while the generated $S_{3}$ component indicates conversion from purely linear polarization to elliptical polarization.

This degradation is particularly harmful in the detector-multiplexing context because the decoder identifies FOV origin by comparing reconstructed Stokes vectors with predefined reference vectors. If the reference vector remains ideal while the measured vector is distorted by the folding mirror, the similarity score is reduced for the correct channel and may increase for neighboring channels. Therefore, even if the optical image is perfectly folded and the detector measurement is noise-free, an uncompensated mirror can introduce a systematic decoding bias in the polarization domain.

**Figure 2:**
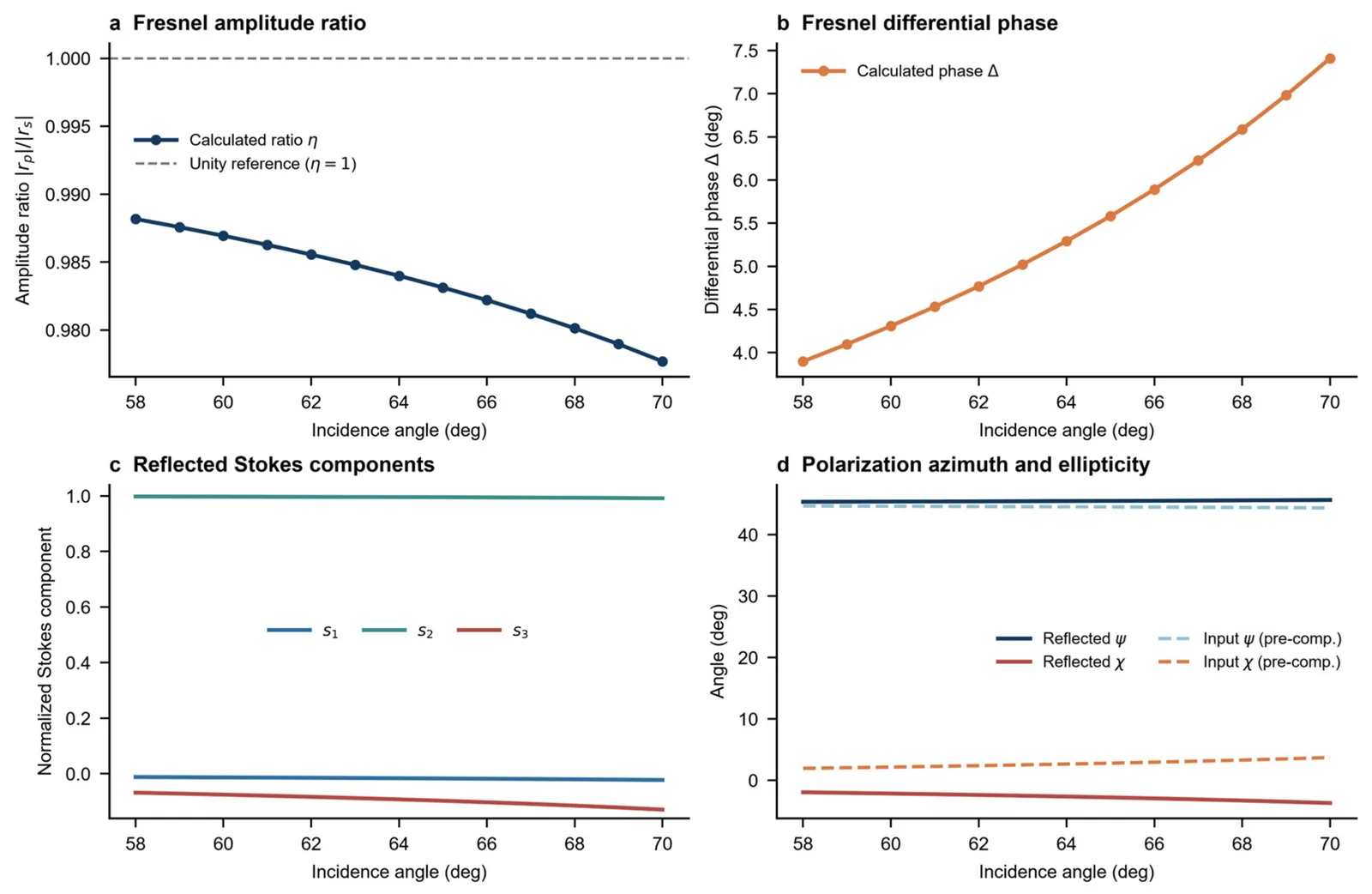
Fresnel mechanism and angle sensitivity for a nominal 45 deg linear code. The upper panels show the relative p/s amplitude and differential phase; the lower panels show the reflected Stokes components and the corresponding azimuth/ellipticity parameters. The change is deterministic in the ideal Jones model and is removed to numerical precision by inverse pre-compensation.

The analytical expressions in Section 2.3 show that the severity of this bias is controlled by eta and Δ. A small amplitude imbalance mainly changes $S_{1}$, while a nonzero differential phase mainly generates $S_{3}$. In practical terms, this means that the mirror can simultaneously shift the polarization azimuth and introduce handedness. Both effects must be considered when designing reference polarization states for a folded optical system. The corresponding Fresnel amplitude ratio, differential phase, reflected Stokes components, and polarization-angle evolution are summarized in Figure 2.

### 4.3. Pre-Compensation Results from Mueller-Matrix Inversion

By enforcing the detector-side target state, the required input states were obtained by inverse propagation. The computed states remain physically valid and fully polarized over the complete 58–70 deg incidence range. Under the common right-handed coordinate convention, the azimuth remains close to +45 deg and the required positive ellipticity increases gradually with incidence angle.

Representative pre-compensation results are listed in Table 3.

After these pre-compensated states pass through the mirror, the reflected output remains close to

(46) $$S_{out}\sim {\left[0.96,\;0,\;0.96,\;0\right]}^{T}$$

over the full angular range. Thus, $S_{2}$/$S_{0}$ remains approximately unity and $S_{3}$/$S_{0}$ remains approximately zero. The reduction of $S_{0}$ below unity represents mirror reflectance loss rather than polarization-code error. The normalized Stokes state is therefore recovered even though the absolute intensity is attenuated.

**Figure 3:**
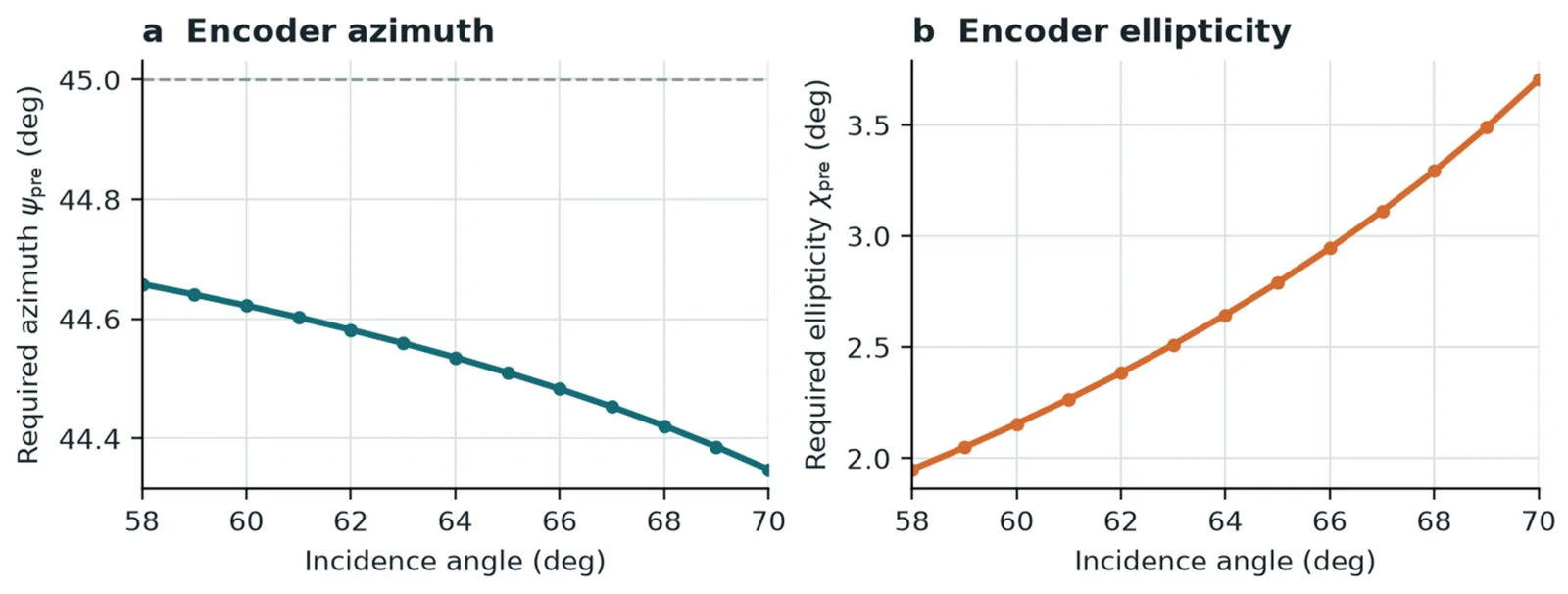
Corrected angle-dependent pre-compensation parameters obtained by inverse propagation. The encoder-side azimuth remains close to +45 deg, while the ellipticity angle increases smoothly with incidence angle under the stated right-handed coordinate and S_3_ convention. The blue curve denotes the required encoder azimuth $\psi_{pre}$, whereas the orange curve denotes the required encoder ellipticity $\chi_{pre}$.

These results indicate that, for a known mirror geometry, the intended 45 deg detector-side code should be generated as a slightly elliptical incident state. Under the stated coordinate convention, the required azimuth changes from 44.66 deg to 44.35 deg and the ellipticity angle increases from approximately 1.95 deg to 3.70 deg over the simulated range. The sign of these parameters depends on the adopted propagation and handedness convention; the full Stokes or Jones vector is the unambiguous implementation quantity. The angle-dependent encoder-side pre-compensation parameters are shown in Figure 3.

### 4.4. LCPR Post-Compensation Results

The LCPR optimization provides a second route for preserving the 45 deg polarization code when the input state is fixed. For each incidence angle, the reflected field was passed through a tunable retarder, and the pair (α, Δ) was selected by minimizing the Jones-domain residual relative to the target 45 deg linear state.

The single-channel parameter trend is summarized in Table 4.

For the isolated 45 deg state, the numerical solutions can be mapped into equivalent branches because retarder settings are periodic. The apparent 90 deg changes in fast-axis orientation are therefore a modulo-periodicity of the optimizer rather than a physical switching requirement. These per-angle values should not be interpreted as settings that one shared post-path LCPR can apply simultaneously to all nine channels.

Compared with pre-compensation, post-compensation has a different practical role. Pre-compensation embeds the mirror response into the polarization encoding stage and is attractive when the polarization mask or modulator can be designed channel by channel. LCPR post-compensation is more flexible when the incident encoded states are fixed or when the optical system must adapt to changes in incidence angle, temperature, or mirror coating. In a practical system, a hybrid approach may be used: static pre-compensation can remove the dominant deterministic distortion, while a tunable retarder can correct residual deviations.

**Figure 4:**
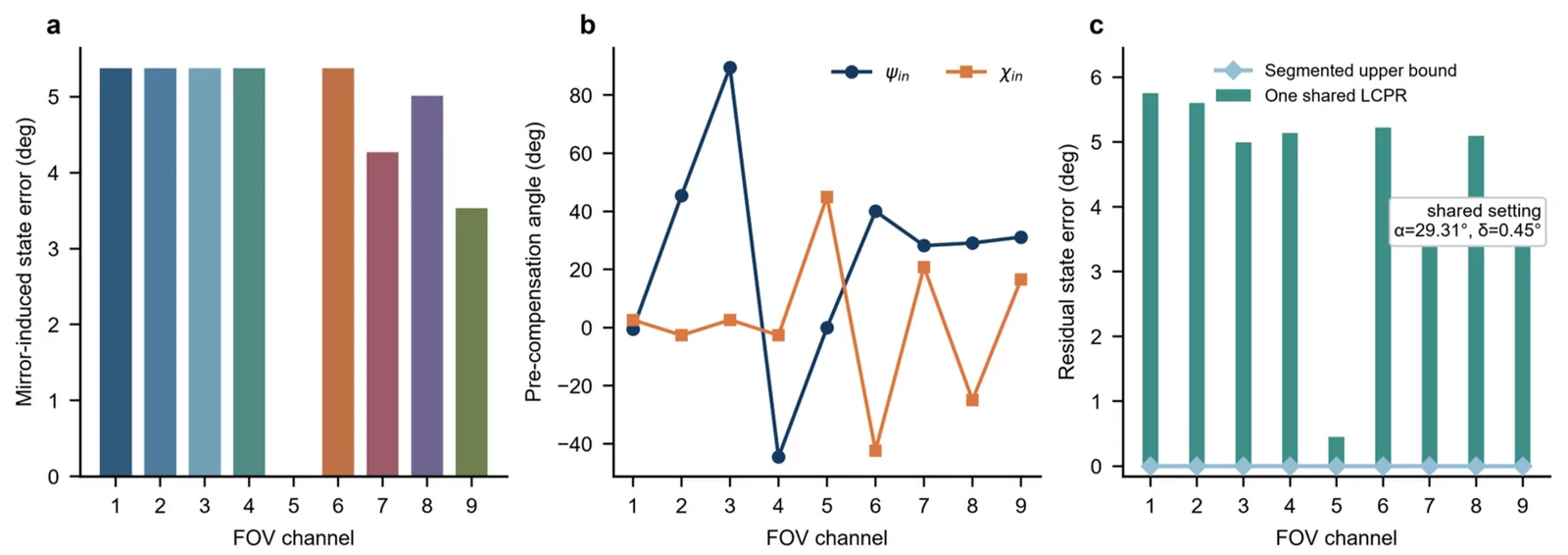
Channel dependence and LCPR implementation boundary. Mirror-induced state displacement and encoder-side pre-compensation are channel-dependent. Independent segmented-LCPR settings provide a state-restoration upper bound, whereas one shared post-path LCPR leaves residual channel errors because the same two control parameters act on all merged channels. Colors in (**a**) distinguish the encoded FOV channels; the blue and orange curves in (**b**) denote $\psi_{pre}$ and $\chi_{pre}$, respectively; and the two bar colors in (**c**) distinguish the one-shared-LCPR result from the segmented-LCPR upper bound.

For the complete nine-channel system, joint optimization gives a shared-LCPR setting close to the identity transformation (fast-axis angle 29.31 deg and retardance 0.45 deg), whereas independent segmented settings can recover individual pure states to numerical precision. This contrast defines the hardware boundary without removing the practical value of LCPR correction in a segmented or sequential architecture. The channel dependence and the implementation boundary between shared and segmented LCPR compensation are illustrated in Figure 4.

### 4.5. Detector-Side Calibration and Code-Space Criterion

The results imply that the reference library used for FOV decoding should be defined after optical propagation, not merely at the encoder plane. For the k th channel, the appropriate detector-side reference vector is

(47) $$S_{k}^{\mathrm{ref}}=M_{\mathrm{sys},k}S_{k}^{\mathrm{enc}}$$

where $M_{\mathrm{sys},k}$ includes all polarization-affecting optical elements in the k th folded path. If compensation is applied, the reference becomes

(48) $$S_{k}^{\mathrm{ref}}=M_{\mathrm{comp},k}M_{\mathrm{sys},k}S_{k}^{\mathrm{enc}}$$

The previous ideal decoder can therefore be generalized without changing its computational structure. The similarity matching still operates in Stokes space, but the reference vectors are calibrated using the full polarization transfer matrix of the optical path. This observation is important because it means mirror compensation can be integrated into the existing polarization-encoded multiplexing pipeline rather than requiring a completely new reconstruction algorithm.

From a system perspective, the most important design requirement is to maintain sufficient angular separation between detector-side reference states. Compensation should therefore be evaluated not only by whether one state is recovered accurately, but also by whether the complete set of FOV codes remains well distributed on the Poincare sphere after folding, reflection, and detection. This provides a direct path for extending the present single-state study to the full nine-region encoding architecture.

### 4.6. Nine-Region Accuracy Under Calibration and Compensation Conditions

To quantify mirror compensation without conflating it with decoder calibration, pixel-level classification accuracy was evaluated under additive Gaussian noise in the six polarization measurement channels. In addition to the original ideal-codebook comparison, a detector-side calibrated codebook is included. The calibrated case uses the full mirror-propagated Stokes references and therefore isolates noise and code-space separation from avoidable model mismatch.

The corrected and expanded accuracy comparison is summarized in Table 5.

With the corrected coordinate-consistent mirror model, the uncompensated ideal-codebook decoder no longer collapses to the previously reported approximately 30% baseline. All evaluated methods remain approximately 100% accurate at σ = 0.02. The earlier low baseline is not interpreted as mirror-induced information loss because it primarily reflected reference-library and coordinate mismatch.

**Figure 5:**
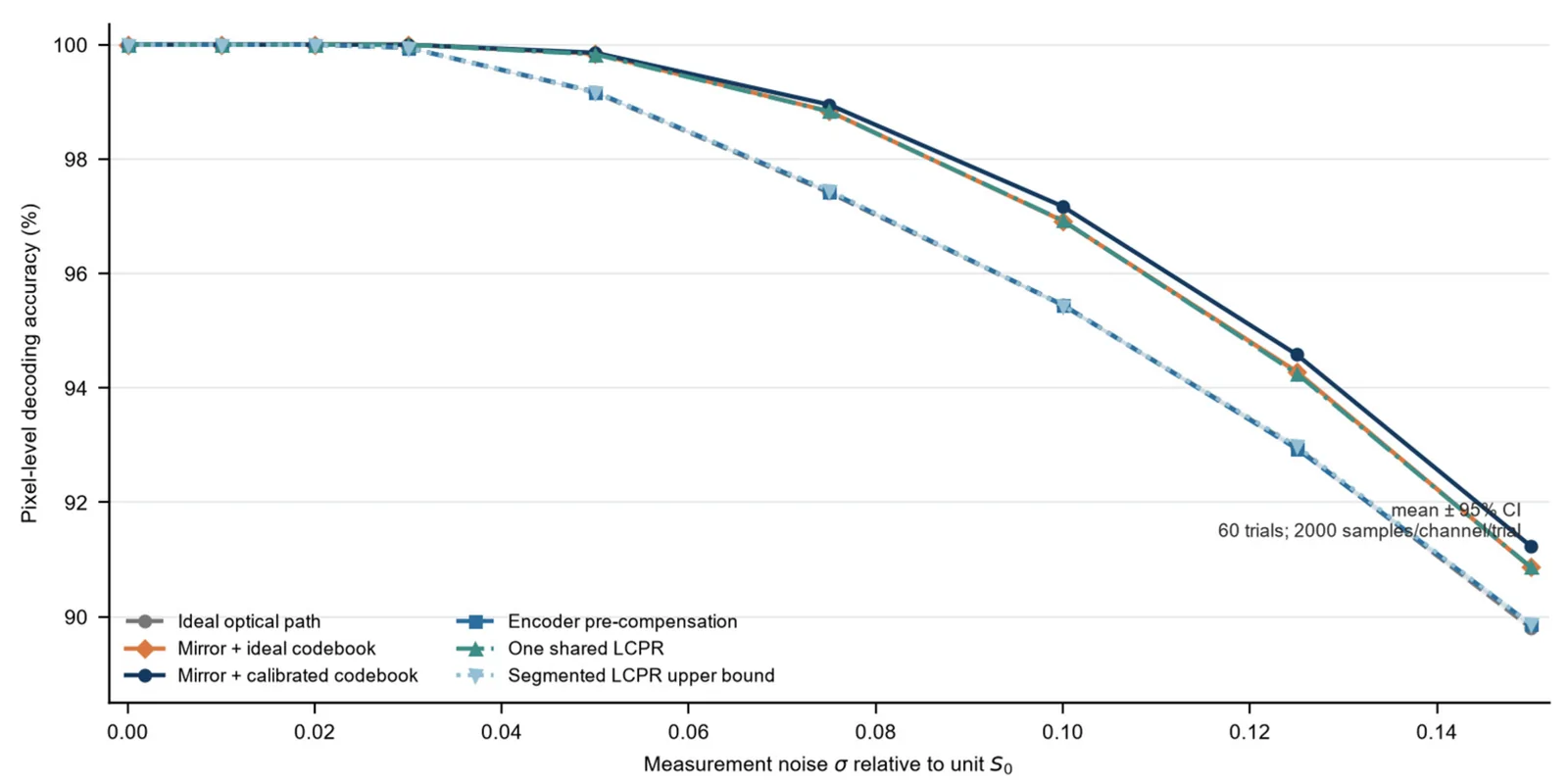
Corrected nine-region decoding accuracy under ideal propagation, mirror transport, detector-side calibration, encoder pre-compensation, one shared LCPR, and a segmented-LCPR upper bound. Lines show means over 60 Monte Carlo trials with 2000 samples per channel per trial; shaded regions show 95% confidence intervals.

Differences become visible at higher noise. At σ = 0.15, detector-side calibration achieves 91.24%, compared with 90.87% for the mirror plus ideal codebook and 89.87% for encoder pre-compensation. The calibrated case performs slightly better because the nominal mirror transformation increases the minimum pairwise separation of this particular nine-state codebook from 15.0 deg to 21.7 deg. Pre-compensation remains valuable when a prescribed detector-side codebook is required, but it is not automatically the most accurate classifier under every noise and geometry condition. The complete noise-dependent classification-accuracy comparison is shown in Figure 5.

## 5. Discussion

The present analysis strengthens the physical foundation of polarization-encoded detector multiplexing by addressing a component that is unavoidable in compact folded infrared systems. The central result is that a folding mirror introduces deterministic polarization distortion through p/s diattenuation and retardance. This distortion is strong enough to alter the Stokes vector of a canonical 45 deg linear code, but it is also structured enough to be modeled and compensated.

The distinction between polarization-code distortion and intensity loss is important. Mirror reflectance reduces $S_{0}$, while polarization degradation changes the normalized vector $\left[\begin{array}{ccc} S_{1} & S_{2} & S_{3} \end{array}\right]/S_{0}$. Detector multiplexing can often tolerate a moderate intensity loss if the signal-to-noise ratio remains sufficient, but it cannot tolerate uncontrolled drift of the normalized Stokes code because FOV identity is carried by that vector. Thus, the normalized polarization state should be treated as a calibrated information carrier in the same way that geometric distortion is calibrated in conventional imaging systems.

The pre-compensation results suggest that a folding mirror can be absorbed into the encoder design. Instead of assigning ideal Stokes states at the modulation plane, one can assign mirror-aware incident states whose detector-side outputs match the desired codebook. This strategy is conceptually elegant but requires that each channel’s incidence angle and mirror coating response be known in advance. It is most suitable for fixed optical layouts with stable thermal and mechanical conditions. The nine-region simulation supports this interpretation quantitatively: once the mirror response is included in the encoder-side state design, the original Stokes-domain decoder recovers high classification accuracy without changing its computational structure.

The LCPR compensation results suggest a more adaptive implementation path. A tunable retarder can restore the polarization state after reflection without redesigning the upstream polarization encoder. The near-half-wave optimal retardance observed in the simulation is favorable because it implies that the compensation map is simple and physically interpretable. Nevertheless, a single global LCPR may be insufficient if different FOV channels encounter substantially different incidence angles or multiple mirrors with different planes of incidence. In that case, channel-wise compensation, segmented retarders, or calibration-based detector-side references may be required.

Temperature drift is an additional implementation concern. Temperature-dependent metal optical constants and, more strongly, LCPR retardance-voltage behavior can change the calibrated transfer matrix and compensation setting. Periodic in situ calibration or a temperature-indexed lookup table is therefore required for a practical infrared instrument [36,37].

Several limitations remain. First, the numerical model assumes an ideal smooth, spatially uniform bulk-aluminum surface; roughness, native oxide, protective layers, coating-thickness nonuniformity, finite bandwidth, and pupil averaging may introduce additional polarization aberration or true depolarization. Second, the regular eight-facet geometry is a system model informed by the optical design rather than a measured prototype. Fabrication would require diamond turning or individually aligned coated facets, with facet-angle, edge, roughness, and coating tolerances included in calibration. Third, real infrared scenes may include partially polarized radiation, detector nonuniformity, alignment errors, and temperature drift. These effects require measured channel-wise Mueller matrices and experimental validation [20,21,35,38].

Despite these limitations, the proposed framework provides the necessary bridge between ideal polarization encoding and physically realistic folded optics. It shows how the mirror matrix can be inserted into the forward model, how source-side and detector-side compensation can be designed, and how the corrected states can be returned to the existing Stokes-domain decoder. This makes the method extensible: additional mirrors, waveplates, coatings, and detector-side polarization analyzers can be included by multiplying their corresponding Jones or Mueller matrices in the optical sequence.

In summary, this work investigated the polarization-state degradation caused by folding mirrors in polarization-encoded detector-multiplexed infrared imaging systems. Starting from the Jones representation of oblique metallic reflection, the mirror was shown to act as a diattenuating retarder whose amplitude ratio and differential phase distort the designed Stokes-vector code. For a nominal 45 deg linear state, the derived expressions show that p/s amplitude imbalance introduces $S_{1}$ and differential phase introduces $S_{3}$, converting the ideal code into an angle-dependent elliptical state.

Two compensation strategies were developed and retained. Inverse propagation provides an angle-dependent pre-compensation state that restores a prescribed detector-side code, while an LCPR provides a tunable post-reflection route. The revised system analysis clarifies that per-channel LCPR solutions require segmented or separated hardware and that a single shared LCPR cannot realize all channel-specific settings simultaneously. Under the corrected common protocol, all methods remain approximately 100% accurate at σ = 0.02; at higher noise, detector-side calibration is competitive because it preserves the transformed code-space separation.

The results demonstrate that folding-mirror polarization effects must be included in the design of polarization-encoded detector-multiplexed infrared imaging systems. At the same time, the distortion is deterministic and can be corrected through calibrated pre-compensation, tunable post-compensation, or mirror-aware detector-side reference vectors. This work therefore advances polarization-domain detector multiplexing from an ideal encoding concept toward a more complete physical model suitable for compact folded infrared optical systems.

## Figures and Tables

**Table 1:** Centralized simulation parameters and reproducibility settings.

Parameter	Value	Parameter	Value
Wavelength	4.0 um	Al optical constant	6.77 + 38.68i
Peripheral incidence	64 deg	Angle sweep	58–70 deg; 1 deg
Noise σ	0–0.15	Signal scale	Uniform 0.75–1.25
Monte Carlo trials	60	Samples	2000/channel/trial
Random seed	4,516,662	Analyzer channels	0, 45, 90, 135, R, L

**Table 2:** Nine-region polarization codebook and nominal folding geometry.

Channel	FOV	Path	theta (deg)	phi (deg)	psi (deg)	chi (deg)
1	NW	Mirror	64	135	0	0
2	N	Mirror	64	90	45	0
3	NE	Mirror	64	45	90	0
4	W	Mirror	64	180	−45	0
5	Center	Direct	0	-	0	45
6	E	Mirror	64	0	0	−45
7	SW	Mirror	64	225	30	22.5
8	S	Mirror	64	270	30	−22.5
9	SE	Mirror	64	315	30	15

**Table 3:** Required incident polarization states for restoration of the 45 deg linear detector-side code.

Incidence Angle (deg)	Required Incident Stokes State [S_0_, S_1_, S_2_, S_3_]	psi (deg)	chi (deg)	DoP
58	[1.000, 0.012, 0.998, 0.068]	44.66	1.95	1.000
59	[1.000, 0.013, 0.997, 0.071]	44.64	2.05	1.000
60	[1.000, 0.013, 0.997, 0.075]	44.62	2.15	1.000
61	[1.000, 0.014, 0.997, 0.079]	44.60	2.27	1.000
62	[1.000, 0.015, 0.996, 0.083]	44.58	2.38	1.000
63	[1.000, 0.015, 0.996, 0.088]	44.56	2.51	1.000
64	[1.000, 0.016, 0.996, 0.092]	44.54	2.65	1.000
65	[1.000, 0.017, 0.995, 0.097]	44.51	2.79	1.000
66	[1.000, 0.018, 0.995, 0.103]	44.48	2.95	1.000
67	[1.000, 0.019, 0.994, 0.108]	44.45	3.11	1.000
68	[1.000, 0.020, 0.993, 0.115]	44.42	3.29	1.000
69	[1.000, 0.021, 0.992, 0.122]	44.39	3.49	1.000
70	[1.000, 0.023, 0.991, 0.129]	44.35	3.70	1.000

**Table 4:** Equivalent per-angle LCPR solutions for the canonical 45 deg input state.

Incidence Angle (deg)	Optimal Retardance ***Δ*** (deg)	Optimal Axis ***α*** (deg)
58	178	122
59	178	122
60	178	122
61	182	32
62	182	32
63	182	32
64	178	124
65	178	124
66	178	124
67	182	34
68	182	34
69	178	126
70	178	126

**Table 5:** Mean nine-region pixel-level classification accuracy under the corrected common simulation protocol.

Method	σ = 0.02	σ = 0.10	σ = 0.15	Implementation
Ideal optical path	100.00	95.45	89.81	Reference
Mirror + ideal codebook	100.00	96.92	90.87	Uncalibrated
Mirror + calibrated codebook	100.00	97.17	91.24	Calibration
Pre-compensation	100.00	95.45	89.87	Per channel
One shared LCPR	100.00	96.93	90.87	Global setting
Segmented LCPR	100.00	95.44	89.87	Per channel

## Data Availability

The data presented in this study are available upon request from the corresponding author.
